# Hidden in visible light: spectral-temporal unmixing of lung tissue autofluorescence in a fibre-based system

**DOI:** 10.1364/BOE.587887

**Published:** 2026-03-31

**Authors:** Alexandra C. Adams, Layla Mathieson, Mark Austin, Liam Neilson, András Kufcsák, Mohsen Khadem, Ahsan R. Akram, Kevin Dhaliwal, Sohan Seth

**Affiliations:** 1 Translational Healthcare Technology Group, Institute for Regeneration and Repair, 5 Little France Dr, Edinburgh, EH16 4UU, United Kingdom; 2 School of Informatics, University of Edinburgh, United Kingdom; 3 Institute of Photonics and Quantum Sciences, Heriot-Watt University, Edinburgh, EH14 4AS, United Kingdom

## Abstract

Autofluorescence molecules, including metabolites and structural tissue components that play a crucial role in cellular processes, are often disrupted during lung cancer oncogenesis. These fluorophores emit photons when they return to their ground state after excitation, where the average time spent in the excited state, i.e., fluorescence lifetime, is influenced by their environment. The fluorescence process is highly sensitive to nuanced environmental changes, making it an ideal method for personalized lung cancer diagnosis. However, current *fibre-based* fluorescence devices typically use broad wavelength channels, and computational methods often compute average fluorescence lifetimes, reducing the sensitivity and specificity for detecting subtle disease changes. We provide evidence that a high-resolution spectral-temporal time-resolved fluorescence spectroscopy (TRFS) device (0.5 nm, 50 ps) coupled with the multichannel fluorescence lifetime estimation (MuFLE) model can unmix underlying individual components label-free using their spectral and temporal characteristics simultaneously. In lung tissue *ex vivo*, we extract paired fluorescence lifetimes and emission profiles, and spatially correlate these measurements with endogenous fluorophores using confocal FLIM and complementary antibody staining. This technique promises label-free real-time tracking of endogenous fluorophores during lung cancer diagnosis, offering a more personalized, precise metabolic and structural assessment during bronchoscopy in real time.

## 1. Introduction

Fibre-based optical technologies that rely on fluorescence lifetime (FL) estimation, such as Time-Resolved Fluorescence Spectroscopy (TRFS) and Fluorescence Lifetime Imaging (FLIm), have shown promise in delineating cancerous tissue from non-cancerous tissue, both *ex vivo* [1,2] and *in vivo* [3–7]. These devices have the potential to greatly improve lung cancer diagnostics by offering a metabolically sensitive, real-time, *in vivo* and cost-effective alternative to biopsies [8–10].

In the absence of *labels* or fluorescently tagged molecules, these devices capture the autofluorescence (AF) of tissue when excited with a monochromatic light of specific wavelength (e.g., 355 nm or 485 nm) [5,11]. AF originates from naturally occurring endogenous fluorophores, e.g., nicotinamide adenine dinucleotide (phosphate) (NAD(P)H), collagen, flavin adenine dinucleotide (FAD), flavin mononucleotide (FMN), riboflavin, elastin, and porphyrin [12, Fig. 2]. Changes in the photophysical properties of these endogenous fluorophores and their relative abundances in various disease states underpin the discriminating potential of FL based devices [13,14]. For example, the relative abundance and photophysical properties of FAD and NAD(P)H (often measured at channels 542-582 nm and 426-446 nm respectively, and known as the *optical redox ratio*) serve as an indicator of the metabolic status of tissue that is frequently deregulated in cancer [14,15].

Existing approaches often estimate the average FL from observed decay traces across a few broad wavelength channels [2,16]. While this provides a rapid overview of cumulative changes in the photophysical properties of endogenous fluorophores, it may limit the ability to detect nuanced biological features that could further enhance cancer differentiation. For example, when measuring FAD emission (with an expected peak emission between 520 nm -530 nm [17]), in the channel 542 −582 nm [14,18], additional fluorophores such as flavin mononucleotide (FMN, peak emission at 530 nm [19]), riboflavin (peak emission at 532 nm [20]), and elastin (emission range: 360 −600 nm [21]) can also be recorded within this range. Moreover, the average FL within these broad emission channels may not capture the finer photophysical variations in disease-disrupted biological processes. For instance, during glycolysis, the FL of FMN increases (bound FL: 4.7 ns), while during oxidative phosphorylation (OXPHOS), it decreases (unbound FL: 1.5 ns) — a photophysical pattern opposite to that of FAD [14].

The potential change in the average FL in cancerous tissue compared to healthy tissue as a diagnostic tool can be difficult to interpret, as it is the consequence of numerous nuanced variations in the photophysical properties of the underlying endogenous fluorophores, their sensitivity to different environmental states, and their relative abundances in a heterogeneous environment characteristic of lung cancer [22]. We argue that a system capable of un-mixing the underlying components can provide detailed and specific information about the structural components (e.g. elastin) and metabolic (e.g. FAD) proteins and their environmental factors [23], enabling improved diagnostic capabilities and facilitating clinical translation of these technologies.

The fibre-based Extensively-Parallel TRFS (EP-TRFS) device, unlike conventional TRFS setups which typically acquire only a limited number of spectral channels, generates high resolution spectral-temporal histogram profiles of 512 wavelength channels with a wavelength bandwidth resolution of 0.5 nm and 1200 temporal channels with a temporal resolution of 50 ps [24]. This allows simultaneously resolving endogenous fluorophores in both the spectral and temporal domains (across a wider wavelength range and at a higher wavelength resolution) using computational tools such as Multi-channel Fluorescence Lifetime Estimation (MuFLE). In addition, the spectral-temporal unmixing potentially enhances the identifiability of the underlying fluorophores, aiding in the discovery and characterization of novel clinically relevant autofluorescence sources, such as previously uncharacterized lysosomal autofluorescence signals [25], whose origins may be better resolved when additional optical properties are available.

We have previously shown that at an excitation of 485 nm, in a fibre-based system, we can unmix exogenous reference fluorophores accurately in both the spectral and temporal domain simultaneously [24]. We have also observed, in a separate study, that in general the shape of the spectral fluorescence lifetime (SFL) in cancerous tissue is more varied than in non-cancerous lung tissue, suggesting an increase in the photophysical complexity of the underlying endogenous fluorophores in a diseased state [11]. However, the origin of the underlying heterogeneity is yet to be explored in more detail.

**Fig. 1.**
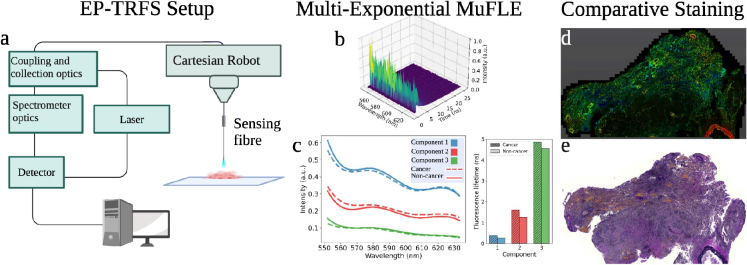
A schematic diagram of the workflow of the paper. **a)** *Ex vivo* lung samples are first assessed using the extensively parallel time resolved fluorescence spectroscopy (EP-TRFS Device) setup. This setup produces high-resolution histograms **b)** which are subsequently analysed by the multichannel fluorescence lifetime estimation model (MuFLE) **c)**. Samples are then prepared for imaging and sliced onto microscope slides before being analysed on the fluorescence lifetime imaging microscope (FLIM) confocal device generating FLIM images **d)**. The results of MuFLE and FLIM are compared. Adjacent slides are finally analysed with a DAPI, collagen and elastin stain **e)**.

In this study, we present preliminary results that high-resolution spectral-temporal measurements from an EP-TRFS device can be deconvolved to reveal individual components, with the focus of this work being the spectral unmixing methodology. We explore this in the context of *ex vivo* lung tissue and observe that the inferred components can be related to fluorophores known to be excited in lung from their fitted spectral (i.e., emission spectrum) and temporal profiles (i.e., lifetime). We observe that there are usually two or three fluorescence components that can be detected in *ex vivo* lung tissue. We validate the temporal profiles of these fluorescence components using FLIm images of a commercial device, and we further validate the presence of the particular fluorescence components using antibody staining of specific biological molecules (i.e., collagen, elastin and nucleus staining ( $4^{\prime }$ -6-diamidino-2-phenylindole or DAPI which binds to double-stranded DNA, thereby highlighting the nucleus)). Figure 1 describes our workflow.

## 2. Methods

### 2.1. Ex vivo lung resections

We utilised fresh resected non-small cell lung specimens *ex vivo* from 28 patients between January 2022 - January 2023 (NHS Lothian BioResource, Scotland Research Ethics Service, reference 15/ES/0094). Samples were collected immediately post resection and stored dry at 4 degrees. Paired samples refer to cancer and non-cancer tissue specimens obtained from the same patient during lobectomy. The paired samples were clinically defined as cancerous and non-cancerous lung samples by a clinical histopathologist. In addition, type, stage and age are also noted (see Table 5). The tissue area available to measure ranged from 50 mm-3 cm. Therefore, depending on the sample size available, between 3-6 locations were measured on the EP-TRFS device. Data collected on the EP-TRFS device was completed within 24 hours of resection, followed by immediate preparation for FLIM data acquisition (see section 2.3). In addition, as mentioned in [11]; measurements were collected using a 3 axis Cartesian robot [26] (see Fig. 1(A)). 3 repeated histograms were summed together, and histograms with a peak FI (fluorescence intensity)(i.e., photon counts) of less than 300 were excluded from the analysis due to poor signal-to-noise ratio. Following assessment on the EP-TRFS device 8 samples were assessed on a FLIM confocal microscope (see section. 2.3 for more detail).

### 2.2. EP-TRFS

The EP-TRFS device used in this study was used in time-correlated single photon counting (TCSPC) mode. The device has been characterised previously in [24] and [11]. A PicoQuant laser, with an excitation of 485 nm (laser diode head (LDH-P-C-485, PicoQuant, Germany) and laser driver (PDL 800-D, PicoQuant, Germany) used at a repetition rate of 20 MHz was used to excite the samples. This bespoke device has a highly integrated high-resolution line sensor which is able to measure 512 spectral channels with a wavelength bandwidth resolution of 0.5 nm and a total of 1200 time bins of 50 ps (temporal) resolution. Histograms of photon arrival times were measured at all channels in parallel using 1.5×10^{6} exposures with a 5 µs exposure time, equating to 7.5 s per histogram, and an average optical laser output power of 175 µW. The fibre has a core diameter of 50 µm and a numerical aperture (NA) of 0.22 (M42L01, Thorlabs). For the purpose of this study, a narrower spectral range was used for AF collection, 160 spectral channels between 552.03 nm-633.12 nm. Limitation to this spectral range exist due to the constraints of the instrument response function (IRF) since an accurate measurement of the IRF is essential for the least squares fitting routine used, and the reference solution used for measuring the IRF has FI in this range.

### 2.3. Fluorescence lifetime imaging microscopy

A Leica STELLARIS Falcon FLIM confocal microscopy setup was used to assess the AF of paraffin embedded lung slides. To prepare the slides, a standard procedure was followed; i.e., the tissue was fixed in formaldehyde 4 % in PBS overnight, then processed for embedding in paraffin on slides, 3 −4 µm thick. The tissue was assessed on the FLIM device using an excitation and emission window matching the EP-TRFS device (i.e., 485 nm for excitation with an emission window of 550 nm-630 nm). A 20x objective lens was used, 512 x 512 spatial pixels were sampled, with a zoom 4 applied, such that image tiles of 145.31 µm x 145.31 µm, with a pixel size of 0.284 µm x 0.284 µm were collected. The analysis was performed using the multi-exponential precise fit method from the Leica X analysis software, whereby a global fit was calculated across the entire image. Then, for each pixel, the intensity-weighted average FL is calculated and displayed using a colorbar matching the FL ranges anticipated to be present from the tissue when analysed using the MuFLE model. The number of exponential components fitted to each FLIM image was matched to the number of components identified by MuFLE analysis of the corresponding EP-TRFS measurement from the same tissue sample, enabling direct validation between the two measurement techniques.

### 2.4. Slide staining

Slides sequential to those analysed by FLIM were stained for elastin and collagen using a modified Verhoeff’s Van Gieson/EVG stain (Abcam, catalogue no. ab150 667) which stained collagen pink, elastin fibres blue/black and nuclei blue/black. Slides were additionally stained with DAPI for nuclear fluorescence imaging. Slides were deparaffinised in xylene and rehydrated in a series of ethanol dilutions and then stained according to manufacturers instructions. Slides were scanned using a Zeiss Axioscan Z1 at 20x magnification. Images were processed and exported using Zen Blue software v2.6.

### 2.5. Multi-exponential MuFLE analysis

We use MuFLE [11,24] to unmix the acquired photon histogram in the spectral and temporal domain simultaneously, where we model each lifetime component as fixed across the emission range. We operate in the multi-exponential setting of the algorithm and fit up to 3 components consistent with the expected number of endogenous fluorophores contributing to tissue autofluorescence at 485 nm excitation (FAD, riboflavin, and elastin) [12], and in line with previous tissue fluorescence lifetime studies [14]. To determine the suitable number of components L>1 present in the tissue data, we fitted MuFLE with 2 and 3 exponentials since we expect three fluorophores to be excited in the tissue data from the excitation wavelength used as described in the literature (see Table. 6). We choose the number of components with the least residual variance, referred to as ℓ (see Supplement 1 Fig. 9). However, we prefer a smaller number of components, i.e., ℓ−1 , if 1) one of the FLs in the best solution is less than 0.1 ns, as this is below the expected range of endogenous fluorophores found in tissue, and 2) if the difference between any two FLs is less than 0.5 ns, as these similar FLs might be a result of convergence issues due to inherently noisy measurements, and they might be originating from the same fluorophore. We use the same hyperparameter settings as described in [11]. Un-paired t tests were used to determine whether lifetime or intensity component values between cancer and non-cancer tissue types were significantly different.

## 3. Results

### 3.1. Simulated data

**Fig. 2.**
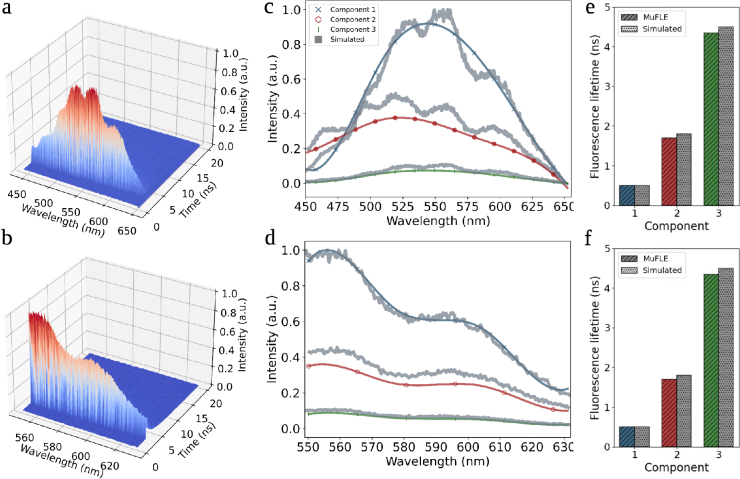
Simulated data comparing full and partial un-mixing with ground truth. **a)** Simulated histogram ranging from 450-650 nm containing the estimated spectral-temporal profiles from three simulated components corresponding to bound and un-bound FAD, in addition to elastin. Spectral noise observed in the EP-TRFS device is also added. **b)** Simulated histogram of the same data in a, however, with a cropped spectral region from 550-630 nm. **c)-d)** Emission profiles un-mixed using multi-exponential MuFLE (solid lines) compared to the simulated emission peaks (variable lines). The simulated spectra and inferred components have been visually aligned. **e)-f)** Fluorescence lifetimes estimated using multi-exponential MuFLE (stripped lines, colored) compared to the lifetimes simulated (dotted, grey). The un-mixed components correspond well to the simulated data representing protein bound FAD (component 1) elastin (component 2) and un-bound FAD (component 3)

We simulated EP-TRFS data using published emission spectra and lifetimes for specific fluorophores present in the lung tissue [27]. First, the accuracy of a multi-exponential MuFLE model is assessed on simulated data. The effectiveness in MuFLE un-mixing simulated fluorophores with overlapping emission spectra and similar FL components are explored (see Fig. 2). In a triple exponential mode, MuFLE accurately un-mixed temporal and spectral profiles of the underlying simulated fluorophores where the spectral regions overlap (as is expected with the fluorophores present in tissue following excitation at 485 nm). Moreover, where complete emission profiles are present, MuFLE accurately un-mixes both the temporal and spectral profiles, showing if this model is to be extended to a broader emission where fluorophore emission peaks are present, MuFLE may also be applied (see Fig. 2).

### 3.2. Multi-exponential MuFLE & tissue

**Fig. 3.**
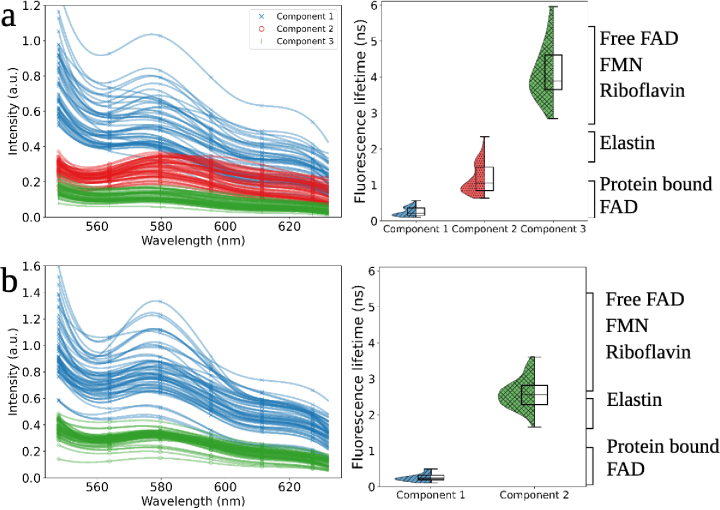
Histograms of *ex vivo* lung tissue measured using a time-resolved fluorescence spectroscopy device, when analysed using the MuFLE model un-mix into three **a)** or two **b)** components. When comparing the fluorescence lifetime values to the anticipated value of endogenous fluorophores from the literature, there is some overlap within the components and protein bound FAD, elastin, free FAD, FMN and riboflavin as shown.

From a total of 28 patients, *ex vivo* lung tissue deemed either cancerous or non-cancerous lung is first assessed with the EP-TRFS device. As aforementioned, the histograms are clipped into the spectral region that contains adequate signal and a robust IRF (comprising 160 wavelength channels from 552.03 nm-633.12 nm and 890 time bins covering 44.5 ns). The signals are then analysed using the multi-exponential mode of MuFLE to recover the spectral-temporal characteristics of the underlying components. From a total of 372 histograms (136 cancerous and 236 non-cancerous lung), 169 histograms is observed to contain 3 components and 185 is observed to contain 2 components. Within the histograms with 3 components, 34 % are cancerous and 66 % are non-cancerous lung (see Fig. 3(A)); within the 2 component data, 36 % are cancerous and 64 % non-cancerous lung (see Fig. 3(B)).

#### 3.2.1. Triple component assessment

The 3 fluorescence components align with FL of potential endogenous fluorophores anticipated to be excited at 485 nm and emit within the measured emission window of 552.03 nm-633.12 nm. Specifically, the FL range of protein bound FAD, as reported in the literature, is anticipated to be between 0.15 ns-0.95 ns [28,29] (see Table. 6). The shortest FL value observed in the tissue data aligns with this FL range (average cancerous 0.38 ns ± 0.25, average non-cancerous lung 0.24 ns ± 0.11) (see Fig. 3(A) & Table 1). The FL of elastin, as reported in the literature, is between 1.72-2.3 ns [29,30] (see Table. 6). In addition, when elastin is measured benchside on the EP-TRFS device, a value of 1.13 ns is observed [11]. The middle FL value estimated within the tissue data aligns well with elastin when measured benchside (average cancerous 1.25 ns ± 0.54, average non-cancerous lung 1.13 ns ± 0.40) (see Fig. 3(A) & Table 1). The longest FL component estimated from the tissue data (average cancerous 4.32 ns ± 1.60, average non-cancerous lung 4.22 ns ± 1.25) (see Fig. 3(A) & Table 1) aligns well with FL values observed from riboflavin on the EP-TRFS device (4.31 ns [11]). Furthermore, similarities are also noted with anticipated FL values of both free FAD and protein bound FMN as reported in the literature (3.13 ns-12 ns) [29,31] (see Table. 6).

**Table 1.** The average fluorescence lifetime of a tri-exponential MuFLE model applied to both cancerous and non-cancerous *ex vivo* lung tissue

	Non-cancerous lifetime (ns)	Cancerous lifetime (ns)	p value
Component 1	0.24 ± 0.11	0.38 ± 0.25	0.046
Component 2	1.13 ± 0.4	1.25 ± 0.54	0.123
Component 3	4.22 ± 1.25	4.32 ± 1.6	0.667

Similarities are observed between the respective emission spectra (see Fig. 3(A)), and when evaluating the relative FI of the 3 components using their corresponding FL and matching emission profiles in both cancerous and non-cancerous lung samples. We observe the highest relative FI, on average, to be consistently originating from the fluorophore with the shortest FL (average cancerous 0.37 ± 0.089, average non-cancerous lung 0.34 ± 0.085). Conversely, the lowest relative FI is observed to originate from the fluorophore with the longest FL (average cancerous 0.11 ± 0.047, average non-cancerous lung 0.11 ± 0.038) (see Supplement 1 Table 7). These findings are consistent with the literature, where it was observed that over 70 % of the emission originated from a short FL component, with the longest FL component displaying the lowest FI 15 %-25 % [14,28].

#### 3.2.2. Double component assessment

Similarities are observed with the component exhibiting the shortest FL value and the FL value of protein bound FAD (average cancerous 0.31 ns ± 0.241, average non-cancerous lung 0.239 ns ± 0.129, see Table 2). Similarities are also observed with the longer FL and the FL corresponding to elastin and the group of flavins (including FMN, unbound FAD, and riboflavin) (average cancerous 2.81 ns ± 0.86, average non-cancerous lung 2.54 ns ± 0.37, see Table 2). Moreover, the FI from the long FL component exhibits the lowest relative FI (average cancerous 0.21 ± 0.05, average non-cancerous lung 0.23 ± 0.033) (see Supplement 1 Table 8), aligning with the findings from the literature and with the 3 component samples. In addition, similarities in the emission profile in both elastin and the group of longer FL flavins can be observed (see Fig. 3(B) & Table 2).

**Table 2.** The average fluorescence lifetime of a bi-exponential MuFLE model applied to both cancerous and non-cancerous *ex vivo* lung tissue

	Non-cancerous lifetime (ns)	Cancerous lifetime (ns)	p value
Component 1	0.23 ± 0.22	0.31 ± 0.24	0.085
Component 2	2.55 ± 0.38	2.81 ± 0.86	0.066

#### 3.2.3. Sample specific un-mixing

**Fig. 4.**
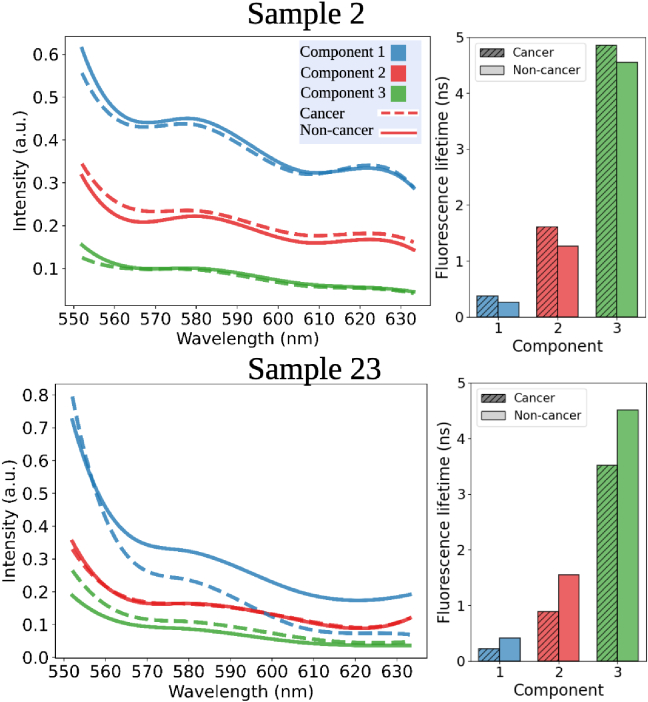
Comparison of cancerous (dashed lines) and non-cancerous lung (solid lines) signals for two samples (2 and 23) reveals patient-specific differences. Un-mixing the fluorescence provides insights into the origins of these distinctions. First, the fluorescence emission profiles between the three un-mixed components differ in both samples. Second, the fluorescence lifetimes of the un-mixed components show distinct variations between cancerous and non-cancerous lung signals.

The spectral-temporal profile of the unmixed components may provide greater insight into sample-specific changes. To illustrate the types of component-specific variations that can occur, we present detailed observations from two representative samples (sample 2 and sample 23) (see Fig. 4 & Table 5). An increase in the average FL (i.e., the FL of the signal from all channels added together) in the cancerous sample compared to the non-cancerous lung sample is observed in sample 2 (average cancerous lifetime: 1.31 ns ± 0.07, average non-cancerous lung lifetime: 1.17 ns ± 0.07). Comparable FI magnitudes is also observed (average non-cancerous lung intensity: 0.44 ± 0.08, average cancerous intensity: 0.49 ± 0.07). When assessing the samples using a multi-exponential MuFLE model, two specific alterations are observed, first, the relative concentration of the 3 components remains similar between both the cancerous and non-cancerous lung samples (see Fig. 4). Second, an increase by 0.34 ns between component 2 (red) and 0.30 ns between component 3 (green) in the cancerous sample, compared to the non-cancerous lung sample is observed (see Fig. 4). We hypothesize that the increase of the FL from these specific components contributes to the overall increase in the cancerous FL compared to the non-cancerous lung FL in this particular instance (see Fig. 4).

Comparable FI magnitudes are observed in sample 23 (average cancerous FI: 0.29 ± 0.15, average non-cancerous lung FI: 0.38 ± 0.15). In addition, comparable average FL values of the cancerous sample compared to the non-cancerous lung sample is also observed (average cancerous lifetime: 1.33 ns ± 0.05, average non-cancerous lung lifetime: 1.32 ns ± 0.04). When assessing the samples using a multi-exponential MuFLE model two sample specific changes can be observed (see Fig. 4). First, the relative concentration of the 3 components between the sample types differs. Most prominently, a change in the relative FI of component 1 (blue) can be observed (the FI corresponding to the FL of 0.22 ns). Particularly, at 600 nm where it is observed to drop below the FI of component 2 (the FI corresponding to the FL of 0.90 ns (i.e., red curve))(see Fig. 4). Second, the non-cancerous lung FL values were higher in the individual components, compared to the cancerous sample (see Fig. 4). We hypothesize that the changes in the relative concentration of the 3 components contribute to the lack of difference between the average FL of the two samples. These two cases illustrate different mechanisms by which component-specific changes can contribute to overall fluorescence lifetime differences between cancerous and non-cancerous lung tissue. However, we note that a systematic analysis of component-specific patterns across the entire dataset would be required to determine the frequency and statistical significance of these different variation patterns across the broader population.

FL values estimated by MuFLE are qualitatively validated using phasor analysis (see Supplement 1 Fig. 10) on two representative samples. For both samples with bi-exponential (A) and tri-exponential (B) decay, respectively, the FL components estimated by MuFLE (black dots) align with the expected phasor positions, confirming the accuracy of the lifetime decomposition. While phasor analysis cannot uniquely decompose mixed signals, the agreement between MuFLE estimates and phasor predictions provides additional validation that the estimated lifetimes are consistent with the measured decay curves.

### 3.3. Autofluorescence images and comparative staining

**Fig. 5.**
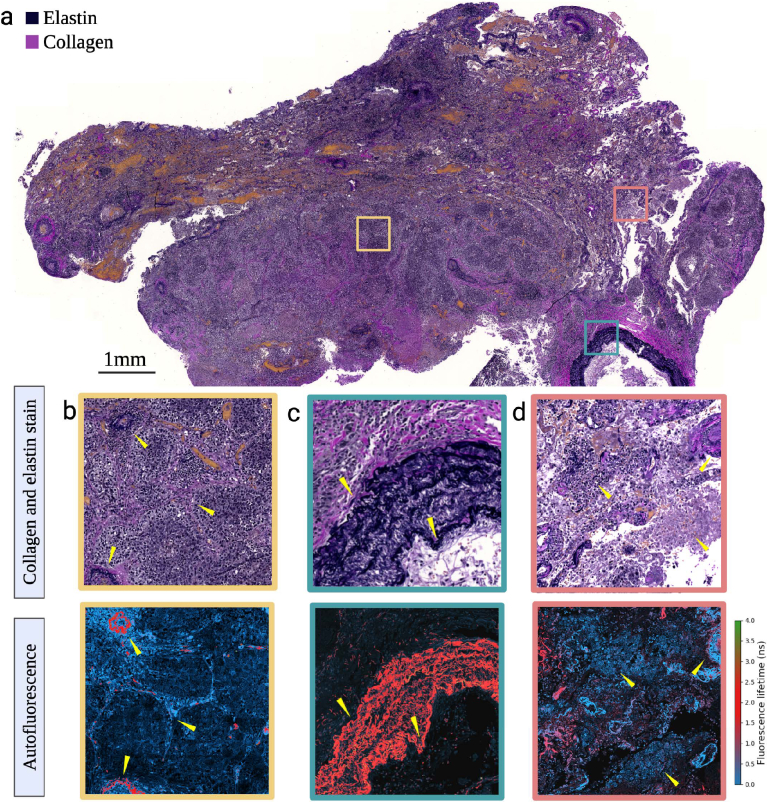
A cancerous lung sample imaged following staining techniques (collagen, elastin & DAPI nuclei staining) and the tissue’s autofluorescence. **a)** The total cancerous lung sample is shown after staining for elastin, collagen and DAPI. **b-d))** Three separate tissue sections (highlighted in yellow, green and red) are shown in more detail. Regions of dense cancer cellularity are visible from the population of packed nuclei (b)). Overlapping elastin stains appear black, indicating regions positive for elastin, where fluorescence lifetimes align with expected elastin signals (green section). Conversely, regions where fluorescence lifetimes correspond to protein-bound FAD (blue) show no clear positive elastin staining (see red and yellow sections).

**Fig. 6.**
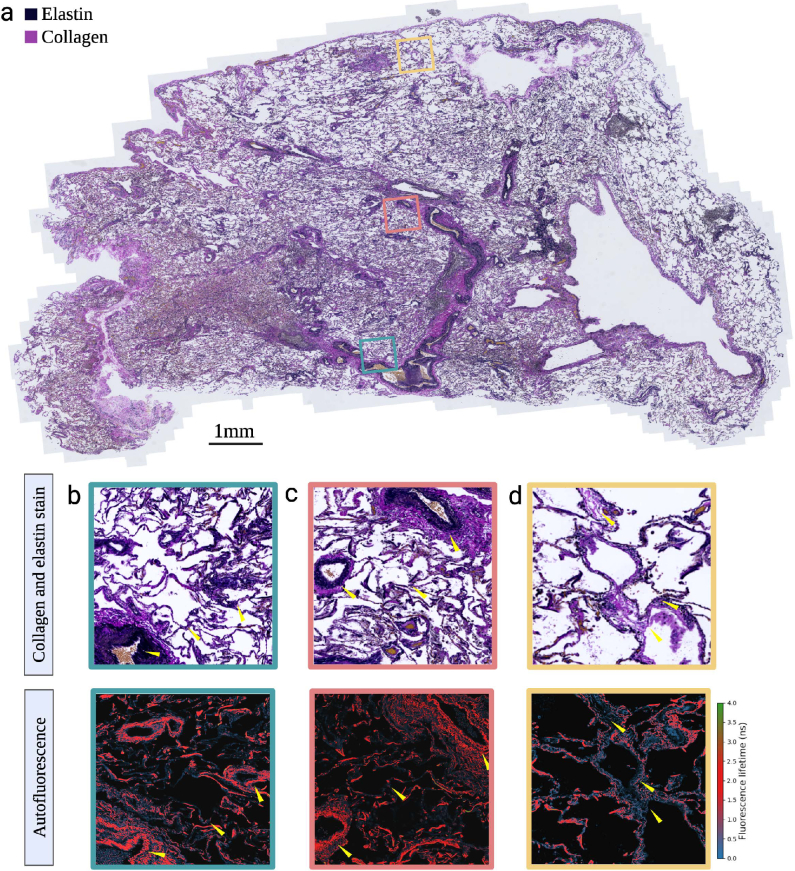
A non-cancerous lung sample imaged using staining techniques (collagen, elastin, and DAPI for nuclei) alongside intrinsic tissue autofluorescence. **a)** The total non-cancerous lung section is shown after staining for elastin and collagen, highlighting structural features of the extracellular matrix. **b-d)** Magnified views of three selected regions (outlined in green, red, and yellow, respectively). Overlapping elastin signals appear black, indicating elastin-positive regions with fluorescence lifetimes consistent with elastin (red). Some areas with significantly shower fluorescence lifetimes (blue, d) show limited overlap with the black elastin stain.

Immediately following assessment on the EP-TRFS device, 8 paired *ex vivo* cancerous and non-cancerous lung samples numbered 11, 15, 16, 24-28, (16 tissue samples in total) are analysed using FLIM confocal microscopy following formalin fixation and paraffin embedded onto slides 10 µm thick. The number of components analysed with FLIM was matched with the number of components when measured with MuFLE, i.e., samples contained either 2 or 3 distinct components (see Section 2.3 & Supplement 1 Fig. 5–6). The FLIM confocal device assesses fluorescence signals from the entire sample, whereas the EP-TRFS device assesses a number of point measurements across the sample (between 3-20 point measurements depending on the tissue size).

In samples where 3 components are observed, the shortest FL value of the components observed in the FLIM device is on average 0.34 ns ± 0.03 in the cancerous and 0.33 ns ± 0.07 in the non-cancerous lung sample (see Table 3), overlapping with the FL measured on the EP-TRFS device of the same samples (see Table 3 & see Supplement 1 Fig. 8). The average value of component 2 within the FLIM data is 1.33 ns ± 0.24 in the cancerous and 1.27 ns ± 0.30 in the non-cancerous lung, also overlapping with the FL of component 2 measured on the EP-TRFS device (see Table 3 & see Supplement 1 Fig. 4). The average value of component 3 observed within the FLIM data is 4.24 ns ± 0.82 in the cancerous and 4.02 ns ± 0.73 in the non-cancerous lung. This value also coincides with the FL of component 3 measured on the EP-TRFS device (see Table 3). In both the cancerous and non-cancerous lung samples, the component with the highest FI had the shorter FL and the component with the lowest FI had the longest FL. This is comparable to the FI from tissue when measured on the EP-TRFS device and analysed using MuFLE (see Supplement 1 Fig. 7 (C) & (D)).

**Table 3.** Tri-exponential MuFLE vs tri-exponential FLIM

	Non-cancerous lung		Cancerous	
	MuFLE (ns)	FLIM (ns)	MuFLE (ns)	FLIM (ns)
Fluorescence lifetime 1	0.264 ± 0.108	0.33 ± 0.071	0.287 ± 0.119	0.344 ± 0.037
Fluorescence lifetime 2	1.24 ± 0.405	1.27 ± 0.305	1.304 ± 0.554	1.337 ± 0.246
Fluorescence lifetime 3	4.464 ± 0.869	4.02 ± 0.730	4.801 ± 2.34	4.239 ± 0.823

In samples where 2 components are observed, the shortest FL value of the components observed in the FLIM device is on average 0.55 ns ± 0.06 in the cancerous and 0.56 ns ± 0.06 in the non-cancerous lung sample (see Table 4). This value is similar to the FL measured on the EP-TRFS device of the same samples (see Table 4). The average value of component 2 within the FLIM data is 2.99 ns ± 0.33 in the cancerous and 3.10 ns ± 0.33 in the non-cancerous lung. The lower bound region of these values (i.e., 2.65 ns in the cancerous and 2.76 ns) are similar to the anticipated FL value of elastin (see Table 4 & 3). In both the cancerous and non-cancerous lung samples, the component with the highest FI had the shorter FL and the component with the lowest FI had the longest FL. This is also comparable to the FI from tissue when measured on the EP-TRFS device and analysed using MuFLE (see Supplement 1 Fig. 7 (C) & (D)).

**Table 4.** Bi-exponential MuFLE vs bi-exponential FLIM

	Non-cancerous lung		Cancerous	
	MuFLE (ns)	FLIM (ns)	MuFLE (ns)	FLIM (ns)
Fluorescence lifetime 1	0.224 ± 0.128	0.560 ± 0.066	0.265 ± 0.130	0.547 ± 0.062
Fluorescence lifetime 2	2.465 ± 0.356	3.104 ± 0.335	2.622 ± 0.406	2.994 ± 0.335

#### 3.3.1. Comparative staining

To examine the spatial distribution of FL from lung tissue, global multi-exponential fitting is applied to the FLIM images measured on the leica confocal. The intesity weighted average FL is then calculated for each image pixel and a colour map corresponding to FL ranges identified using the MuFLE model is applied. This provides subcellular pixel resolution (i.e., 1.4 nm) of the average FL (see Fig. 5 autofluorescence & Fig. 6 autofluorescence). Shortest FL appears to be present in cell like features (see Fig. 5 autofluorescence). Whereas structural features appearing longer in FL are also visible in both the cancerous and non-cancerous lung samples (see Fig. 5 autofluorescence).

To validate the longer FL with elastin, adjacent slides were stained for elastin (black) and collagen (pink) (see Fig. 5 collagen and elastin stain & 6 collagen and elastin stain). Areas which appeared positive for the elastin stain in both the cancerous and non-cancerous lung sample matched bright areas of the longer FL value in all samples (red). Whereas areas which appeared positive for collagen appeared with little to no FI in all samples (see Fig. 5 & Fig. 6). Moreover, in some cancerous tissue when assessed on the FLIM, areas of tissue that has no structural proteins present, an elevated level of fluorescence from short FL is observed (see Fig. 5(d)). In summary, we observe short FL to appear in cellular structures of tissue. In addition, we observe the longer FL value to appear in structural features of tissue, co-localising to the elastin stain, thereby confirming its origin of elastin structures within the lung.

## 4. Discussion

Current devices capable of measuring FL *in vivo* typically assess average FL using channels with low wavelength bandwidth resolution (e.g., [32]). However, understanding the origins of total fluorescence from individual endogenous fluorophores is complex. High-resolution devices, paired with bespoke spectral-temporal analysis tools, may offer deeper insights. Our initial results using multi-exponential MuFLE observe that FL from *ex vivo* lung tissue un-mixes into two or three components in both cancerous and non-cancerous lung samples. These components overlap with expected FLs from fluorophores, such as protein-bound FAD, elastin, and free FAD/FMN/riboflavin, excited by 485 nm. FL is inherently sensitive to local environmental conditions including pH, temperature and protein binding states [33]. The tissue microenvironment in ex vivo lung samples may differ from controlled conditions which naturally leads to FL variations [11]. Moreover, the FL ranges reported in the literature vary considerably between studies due to differences in measurement conditions, including excitation/emission windows, detector systems, and tissue preparation methods (see Table 6). Our observed FL values, while not perfectly matching literature means, fall within biologically reasonable ranges for the identified fluorophores. Thus, we argue that the components observed in our data, whilst not explicitly matching the values found in the literature, fall within an acceptable range, and they are validated by: (1) consistency between EP-TRFS and FLIm measurements, (2) spatial correlation with elastin immunostaining, and (3) simulated data demonstrating MuFLE’s accuracy in unmixing overlapping components.

Specific changes in the average FL and multi-exponential components between cancerous and non-cancerous lung samples are compared in detail (see section 3.2.3). In the first sample, while the relative concentration of the three components remains constant, an increase in the FL of components 2 & 3 contributes to the overall increase in cancerous FL. In the second sample, there is no change in average FL between the non-cancerous lung and cancerous tissue. However, when analysing individual components, we observe an increase in FL across all components in the non-cancerous lung sample, along with a change in the relative concentration of component 1 in the cancerous sample. This shift influences the average FL, so despite a general decrease in FL across all components, the drop in the relative concentration of component 1 raises the overall average FL in the cancerous sample. This counteracts differences observed when only average FL is considered (see section 3.2.3). A different combination of amplitude and lifetime components may also influence the directional change of the average lifetime. E.g., if when comparing cancerous and non-cancerous lung tissue, if the cancerous tissue has higher individual component lifetimes, but a higher amplitude of the shortest lifetime component compared with the other lifetime components, the average lifetime will be shorter. Further highlighting a limitation in average FL analysis. However, a limitation of this specific analysis is that only two cases were analysed in this manner. Future studies should systematically analyse component-level variations across larger sample sizes to establish the prevalence and statistical significance of the patterns we observed. Whilst our analysis is an example of how this can happen, these results highlight how high-resolution models like MuFLE reveal detailed fluorescence properties, which are otherwise lost when relying on average FL of the signal.

We then assess the same tissue using both the EP-TRFS and the Leica STELLARIS Falcon FLIM confocal microscope. We acknowledge differences in tissue processing methods required for data collection between the EP-TRFS device (ex vivo tissue) and FLIM confocal microscope (formalin fixed parafin embedded or FFPE sliced tissue) may alter the fluorescence components. However, studies have shown that whilst the absolute fluorescence signatures may change, the relative fluorescence signatures and component relationships are preserved after fixation [34]. Moreover, our validation emphasizes structural autofluorescence (specific elastin), and the computational un-mixing method makes the approach less vulnerable to specific metabolic artifacts. Furthermore, the FL components identified in samples with three components on the EP-TRFS match those measured on the FLIM device (see Fig. 3(c)). Similarly, FL from samples identified with two components on the EP-TRFS also correspond to those measured on the FLIM device.

Finally, we assess the same tissue with specific staining for collagen, elastin and DAPI. We find that areas positive for elastin correspond to the middle FL value. Additionally, morphologically dying areas correspond to bright regions of FI and the lowest FL value. This feature, an increase in FI during cell death, has been noted in other studies [35,36], however further experiments are needed to confirm the exact origin of this emission. Although we observe FL components linked to cellular features, further analysis, such as metabolic assessments, is needed to confirm whether the observed changes originate from protein-bound FAD, as FL is sensitive to multiple factors (including environmental).

There are several limitations to the EP-TRFS and MuFLE setup used in this study, a major one being the spectral width of the optical system limiting the ability of the device to capture the emission peaks of the anticipated fluorophores, and the number of fluorophores excited at 485 nm. An excitation in the UVA range, such as 355 nm, would likely excite more endogenous fluorophores. Expanding the device’s spectral range to capture more emission peaks would enable a more comprehensive analysis of relative FI (or total spectral emission profiles). Nonetheless, we demonstrate on simulated data that MuFLE accurately extracts individual components even with a larger number of emission channels (see Fig. 2) and maintains accuracy when only a subset of the spectral range is present, even with overlapping components (see Fig. 2). However, the observed histograms from the EP-TRFS are noisy, likely due to the detector’s spectral response (i.e., differences in photon sensitivity between individual SPADs) [37]. These artifacts are not accounted for in the MuFLE model, which may limit the reliability of component assessment in future studies. MuFLE assumes that the effect of dark count is additive and fixed over different time bins, the individual emission spectra are relatively smooth, which can be approximated by B-splines, and the instrument function and maximum number of fluorophores are known relatively well. Violation of this assumption can introduce bias in the estimated parameters. Moreover, another limitation of this study is within the different tissue processing methods applied when measuring tissue on the EP-TRFS device compared to the FLIM Confocal device. Specifically, the FFPE may alter the properties and presence of the fluorophores making direct device comparisons difficult. However, although we observe difference in the FL of the components, the relative concentrations of the components remain the same, suggesting that whilst the FL may differ, the ratios are fixed. All malignant samples were grouped to demonstrate the technical feasibility of the unmixing approach; future studies with homogeneous tumor type cohorts will be necessary to establish subtype-specific biological signatures. The primary focus of this study has been to demonstrate the technical feasibility of an unmixing approach, and therefore, we have pooled samples from different tumour types. A potential future direction of research will be to establish subtype-specific biological signatures, including correlating EP-TRFS measurements with co-registered histopathology to determine how fluorophore contributions vary across distinct tissue microenvironments such as necrotic, hypoxic, fibrotic, and parenchymal regions.

MuFLE assumes that the components have a fixed spectral FL, but in biological systems, due to FL’s sensitivity, determining whether these signals are fixed and originate solely from endogenous fluorophores is challenging. By jointly comparing the lifetime and spectral properties of the components in the sample between endogenous fluorophores when measured benchside, and of the same samples measured on a FLIM device, in addition to comparing the spatial location of these endogenous fluorophores using antibody staining, we can increase the confidence that what we observe is of biological relevance. These results highlight the biophysical detail between cancerous and non-cancerous lung samples with fluorescence decays, which may be un-mixed when high resolution devices, coupled with bespoke analysis models are applied to tissue data, improving both our understanding of AF, and of the diseased tissue.

## 5. Conclusion

In conclusion, we hypothesize that using high-resolution temporal-spectral EP-TRFS devices, coupled with a bespoke analysis model for both temporal and spectral components, allows for the recovery of distinct endogenous fluorophores label-free. This study demonstrates that fluorescence from *ex vivo* lung components, when assessed via a multi-exponential MuFLE model, corresponds to individual components measured benchside and within the literature. The results from paired cancerous and non-cancerous lung samples reveal that the individual components responsible for changes (or lack thereof) in the average FL calculation, are recoverable using this un-mixing method. Additionally, these FL values are consistent when measured on a FLIM device, enabling the spatial localization of individual components. These findings were obtained using a fiber-based device suitable for *in vivo* use. As technology advances, the ability to assess and track specific molecules label-free *in vivo* will improve, potentially enhancing TRFS diagnostic certainty. Thus, integrating this device with MuFLE for *in vivo* applications could enable the real-time measurement of specific molecular and environmental properties tied to endogenous fluorophores in tissue.

## Supplemental information

Supplement 1 Supplemental Document https://doi.org/10.6084/m9.figshare.31760398

## Data Availability

Data presented here is available upon request.

## Supplemental document

See Supplement 1 for supporting content.
