# Supplementary material for: Hidden in visible light: spectral-temporal unmixing of lung tissue autofluorescence in a fibre-based system
Source: Biomed Opt Express. 2026 Mar 31;17(4):2176–90. doi: 10.1364/BOE.587887 (PMC13155838; doi:10.1364/BOE.587887)
Supplement: Supplementary file 1 [file boe-17-4-2176-s001.pdf]

Supplemental Document

Biomedical Optics EXPRESS

Hidden in visible light: spectral-temporal unmixing of lung tissue autofluorescence in a fibre-based system: supplement

ALEXANDRA C. ADAMS,[1] LAYLA MATHIESON,[1] MARK AUSTIN,[2] LIAM NEILSON,[1] ANDRÁS KUFCSÁK,[3] MOHSEN KHADEM,[1] AHSAN R. AKRAM,[1] KEVIN DHALIWAL,[1] AND SOHAN SETH[1,2,*]

[1]*Translational Healthcare Technology Group, Institute for Regeneration and Repair, 5 Little France Dr, Edinburgh, EH16 4UU, United Kingdom*
[2]*School of Informatics, University of Edinburgh, United Kingdom*
[3]*Institute of Photonics and Quantum Sciences, Heriot-Watt University, Edinburgh, EH14 4AS, United Kingdom*
[*]*sseth@staffmail.ed.ac.uk*

This supplement published with Optica Publishing Group on 31 March 2026 by The Authors under the terms of the Creative Commons Attribution 4.0 License in the format provided by the authors and unedited. Further distribution of this work must maintain attribution to the author(s) and the published article's title, journal citation, and DOI.

Supplement DOI: https://doi.org/10.6084/m9.figshare.31760398

Parent Article DOI: https://doi.org/10.1364/BOE.587887

HIDDEN IN VISIBLE LIGHT: SPECTRAL-TEMPORAL UNMIXING OF LUNG TISSUE AUTOFLUORESCENCE IN A FIBER-BASED SYSTEM: SUPPLEMENTAL DOCUMENT

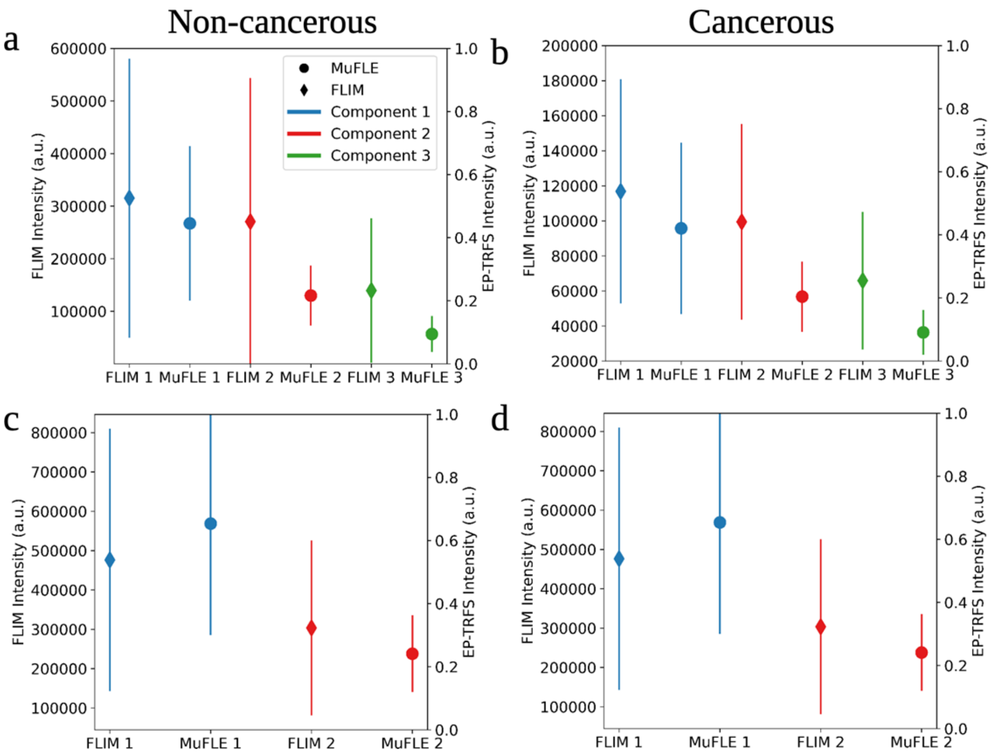

Fig. 1. a and c, non-cancerous triple or double component averages estimated using EP-TRFS, ex vivo tissue and MuFLE compared to FLIM. b and d, cancerous triple or double component averages estimated using EP-TRFS, ex vivo tissue and MuFLE compared to FLIM.

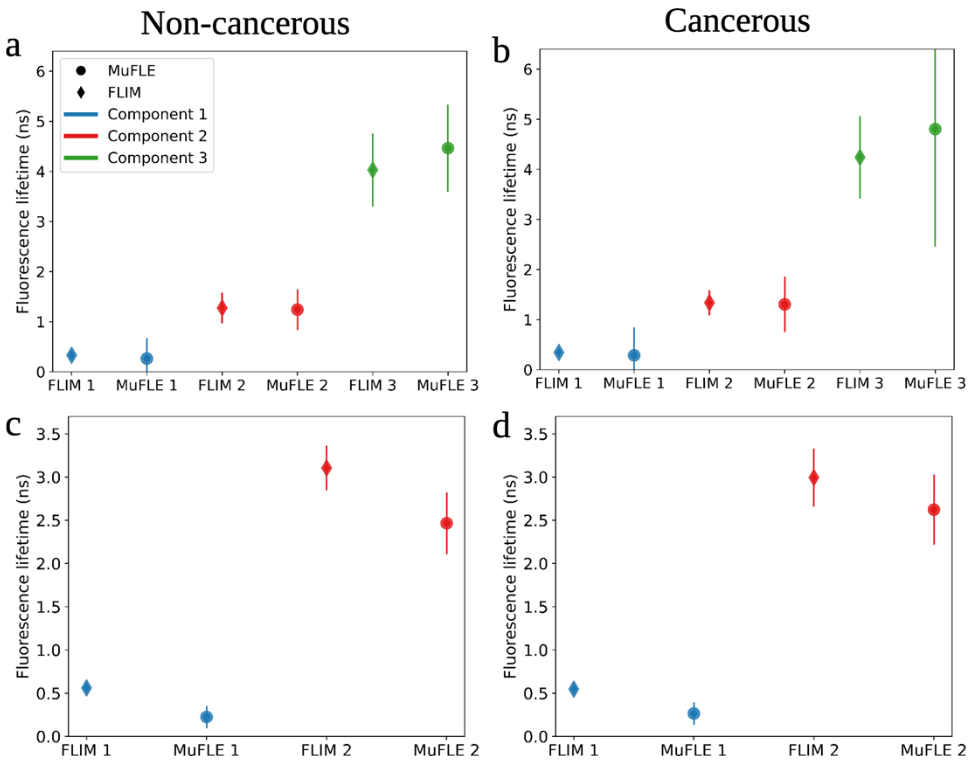

Fig. 2. a and c, non-cancerous triple or double component averages estimated using EP-TRFS, ex vivo tissue and MuFLE compared to FLIM. b and d, cancerous triple or double component averages estimated using EP-TRFS, ex vivo tissue and MuFLE compared to FLIM.

Table 1. Tissue samples analysed alongside their respective features.

Sample number	Cancer type	Stage	Age	Sex
1	Adenocarcinoma	1	63	F
2	Adenocarcinoma	1	63	M
3	Adenocarcinoma	2	73	F
4	Adenocarcinoma	1	74	F
5	Adenocarcinoma	2	77	M
6	Adenocarcinoma	3	86	M
7	Adenocarcinoma	n/a	83	F
8	Squamous cell carcinoma	3	77	M
9	Squamous cell carcinoma	3	66	M
10	Squamous cell carcinoma	3	67	M
11	Squamous cell carcinoma	3	68	F
12	Squamous cell carcinoma	2	77	F
13	Large cell neuroendocrine	3	81	M
14	Malignant melanoma	n/a	83	M
15	Adenocarcinoma	3	53	F
16	Squamous cell carcinoma	1	69	F
17	Adenocarcinoma	3	73	F
18	Squamous cell carcinoma	3	79	M
19	Squamous cell carcinoma	2	82	M
20	Adenocarcinoma	3	67	M
21	Squamous cell carcinoma	2	72	M
22	Adenocarcinoma	3	75	F
23	Adenocarcinoma	3	72	M
24	Squamous cell	1	68	F
25	Squamous cell	2	77	F
26	Adenocarcinoma	1	89	M
27	Squamous cell	1	56	M
28	Pleomorphic carcinoma, giant cell subtype with adenocarcinoma	3	76	F

Table 2. Fluorescence lifetime values of the endogenous fluorophores excited at 485 nm as described in the literature.

Protein bound FAD lifetime (ns)	Elastin lifetime (ns)	Free FAD lifetime (ns)	FMN lifetime (ns)	Riboflavin lifetime (ns)
0.15-0.33 [29]	1.72-1.83 [30]	3.13 [31]	4.27-4.67 [29]	4-12 [29]
0.3-0.4 [28]	2.3 [29]	2.5-2.8 [28]		
0.5-0.95 [29]				

Table 3. The average fluorescence intensity of a tri-exponential MuFLE model applied to both cancerous and non-cancerous *ex vivo* lung tissue.

	Non-cancerous lung intensity (a.u.)	Cancerous intensity (a.u.)	[illegible]
Intensity 1	0.399 ± 0.096	0.382 ± 0.094	0.270
Intensity 2	0.215 ± 0.037	0.199 ± 0.051	0.037
Intensity 3	0.120 ± 0.043	0.106 ± 0.042	0.046

Table 4. The average fluorescence intensity of a bi-exponential MuFLE model applied to both cancerous and non-cancerous *ex vivo* lung tissue.

	Non-cancerous lung intensity (a.u.)	Cancerous intensity (a.u.)	p value
Intensity 1	0.603 ± 0.136	0.524 ± 0.148	0.006
Intensity 2	0.232 ± 0.033	0.218 ± 0.055	0.131

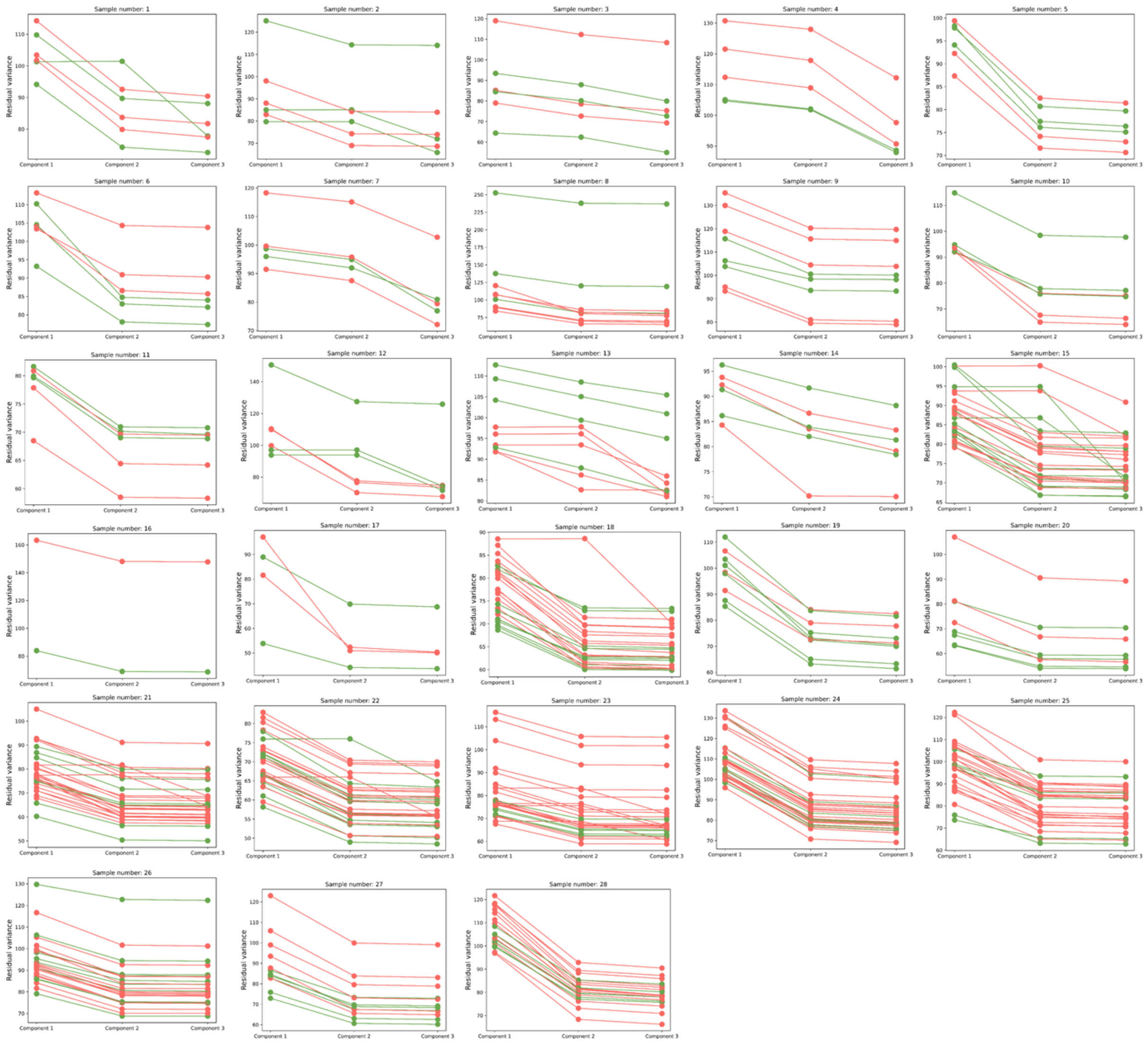

Fig. 3. Plots to show the residual variance of all paired non-cancerous (green) and cancerous (red) *ex vivo* lung samples when analysed on the MuFLE computational models with increasing number of exponentials.

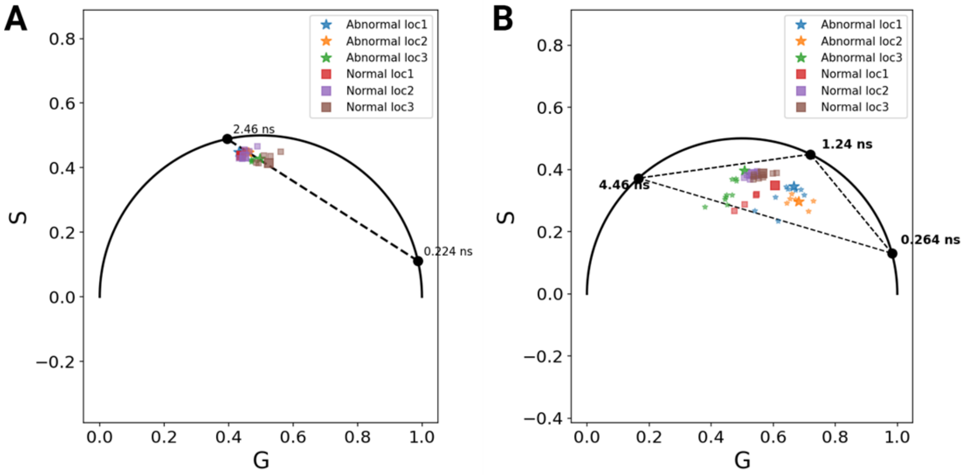

Fig. 4. Phasor plots of two representative samples of **A)** a sample with a bi-exponential decay and **B)** a sample with a tri-exponential decay. Black dots represent the average fluorescence lifetime components obtained from MuFLE analysis of the full dataset, corresponding to the bi-exponential (A) and tri-exponential (B) decay models.
